# A novel compound heterozygous variant of the *SLC12A3* gene in Gitelman syndrome pedigree

**DOI:** 10.1186/s12881-018-0527-7

**Published:** 2018-01-29

**Authors:** Yixin Chen, Ziyi Zhang, Xihua Lin, Qianqian Pan, Fenping Zheng, Hong Li

**Affiliations:** Department of Endocrinology, Zhejiang University School of Medicine Sir Run Run Shaw Hospital, 3 East Qing Chun Road, Zhejiang, Hangzhou 310016 China

**Keywords:** Gitelman syndrome, *SLC12A3*, Compound heterozygous, Pedigree

## Abstract

**Background:**

Gitelman syndrome (GS) is an autosomal recessive disorder caused by genic mutations of *SLC12A3* (Solute carrier family 12 member 3), which encodes the Na-Cl cotransporter (NCC), and presents with characteristic metabolic abnormalities, including hypokalemia, metabolic alkalosis, hypomagnesemia, and hypocalciuria. In this study, we report a case of a GS pedigree, including analysis of GS-associated gene mutations.

**Methods:**

We performed next-generation sequencing analysis and Sanger sequencing to explore the *SLC12A3* mutations in a GS pedigree that included a 35-year-old female patient with GS and five family members within three generations. Furthermore, we summarized their clinical manifestations and analyzed laboratory parameters related to GS.

**Results:**

The female proband (the patient with GS) presented with intermittent fatigue and transient periods of tetany, along with significant hypokalemia, hypomagnesemia, and hypocalciuria. All other members of the pedigree had normal laboratory results without obvious GS-related symptoms. Genetic analysis of the *SLC12A3* gene identified two novel missense mutations (c.1919A > G, p.N640S in exon 15; c.2522A > G, p.D841G in exon 21) in the patient with GS. Moreover, we demonstrated that her mother, younger maternal uncle, and cousin were carriers of one mutation (c.1919A > G), and her father was the carrier of the other (c.2522A > G).

**Conclusion:**

This is the first report of these two novel pathogenic variants of *SLC12A3* and their contribution to GS. Further functional studies are particularly warranted to explore the underlying molecular mechanisms.

**Electronic supplementary material:**

The online version of this article (10.1186/s12881-018-0527-7) contains supplementary material, which is available to authorized users.

## Background

Gitelman et al. first described Gitelman syndrome (GS) as an autosomal recessive disorder presenting with characteristic metabolic abnormalities [1], including hypokalemia, metabolic alkalosis, hypomagnesaemia, hypocalciuria, and renin-angiotensin-aldosterone system (RAAS) activation, along with normal or low blood pressure. GS is usually caused by inactivating mutations of *SLC12A3* (OMIM, 600,968), located on chromosome 16q13, which encodes the thiazide-sensitive Na-Cl cotransporter (NCC) in the renal distal convoluted tubule (DCT).

The clinical manifestations of GS can be extremely variable, most of which are asymptomatic or mild. It was proposed that GS could be one of the main reasons for low blood pressure in the Japanese population, in which the overall frequency of GS mutations was estimated as 0.0321. Therefore, it was reasonable to expect one subject carrying a heterozygous GS mutation among 15.6 Japanese or 10.3 GS subjects among 10,000 Japanese [2]. Additionally, Tamaro [3] stated that GS should be taken into consideration when treating infants with unexplained hypokalemia. A long-term follow-up of one patient who suffered from GS since she was 2.5 years old revealed that it was possible to obtain satisfactory growth and physical development with proper treatment [4]. However, it was argued that patients with early-onset and severe manifestations could suffer from growth retardation [5]. Patients with GS exposed to continuous hypovolemia and hypokalemia had an elevated risk of chronic kidney disease, and to date, two patients with GS have been reported as suffering from end-stage renal failure worldwide [6, 7]. Therefore, it is important for these patients to be recognized early, diagnosed, and treated. Currently, a diagnosis of GS can only be obtained by genetic analysis.

In the present study, we summarize the clinical manifestations, laboratory examinations, and *SLC12A3* gene status of a GS pedigree.

## Methods

### Patient recruitment and clinical evaluation

A 35-year-old female with a previous medical history of hypertrophic cardiomyopathy (HCM) for 15 years was hospitalized to evaluate intermittent fatigue, transient periods of tetany for over 20 years, and accidentally discovered hypokalemia for 3 years. For 20 years, the patient tired easily. In addition, she complained of intermittent self-remitting tetany that occurred after prolonged activity and usually lasted for 5–20 min. Furthermore, she denied other detrimental neuromuscular symptoms (e.g., repeated episodes of palsy, hypotonia, and blurred vision). Three years ago, she was found to suffer from hypokalemia (her plasma potassium was 2.7 mmol/L) after a routine physical examination. She has required treatment with K^+^ supplements since then, during which period her minimum plasma potassium level reached 2.4 mmol/L. However, her hypokalemia was not properly managed via intravenous K^+^ supplements, for which reason she was admitted to our hospital.

The patient took Betaloc for over 5 years to treat the HCM, but stopped taking the medicine when she prepared for pregnancy. However, she miscarried several times for unknown reasons and currently has no children. The family history indicates that the patient’s parents are not consanguineous. Her sister suffered from sudden death when she was 22 years old. Her mother and two maternal uncles have been diagnosed with hypertrophic cardiomyopathy for many years.

On examination, the patient was conscious. Her blood pressure was 114/76 mmHg and her heart rate was 110 beats per minute, as measured with a digital electronic sphygmomanometer. Her respiratory rate was 20 breaths per minute and temperature was 36.0 °C. A 3/6 systolic blowing murmur was heard at the second intercostal space along the left sternal border. Her muscle tone, power, and deep tendon reflexes were normal. The results of other physical examinations were negative.

After admission, we monitored her blood pressure twice daily, which showed that the systolic blood pressure was within the range of 94–114 mmHg, whereas the diastolic blood pressure was within 55–70 mmHg. Electrocardiographic and echocardiographic evaluations were conducted for the patient. She also undertook an adrenal enhanced computed tomography (CT) scan.

The patient’s parents, two maternal uncles, and cousin were also evaluated for clinical manifestations and biochemical abnormalities (Additional file 1: Table S1). All pedigree members had normal blood pressure without any GS-related symptoms. In addition, physical examinations were unremarkable. Laboratory parameters of all members were determined by the clinical laboratory in Sir Run Run Shaw Hospital, Hangzhou, Zhejiang, China.

### Screening for mutations in *SLC12A3*

To define the genetic defect of the patient, and to confirm the *SLC12A3* gene status of this pedigree, sequencing of the *SLC12A3* gene was performed in the index case and her relatives. All of the studies were accomplished in the Zhejiang California International Nanosystems Institute.

#### Samples

A peripheral blood sample (2 ml) was collected from each subject. Genomic DNA was isolated from the peripheral blood using a TIANamp Genomic DNA Kit (TIANGEN, Hangzhou, China) according to the manufacturer’s instructions.

#### Gene amplification

Thirty-three primer pairs were designed to amplify the coding sequence of *SLC12A3* (Additional file 2: Table S2). All the primers were designed by the Zhejiang California International Nanosystems Institute and then purchased from Thermo Fisher Scientific Inc. (Shanghai, China). Polymerase chain reaction (PCR) was performed using Q5 High-Fidelity DNA Polymerase (New England Biolabs, Hangzhou, China). Each reaction contained the following: Q5 high-fidelity DNA polymerase 1 μl (0.5 U/μl), dNTP (2.5 mM) 2 μl, forward primer (2 μM) 5 μl, reverse primer (2 μM) 5 μl, 5 × Q5 buffer 5 μl, template DNA (50–200 ng) 1 μl, and nuclease-free water 6 μl. The cycle conditions were: predenaturation at 98 °C for 30 s; followed by 30 cycles involving denaturing at 98 °C for 8 s, annealing at 57–60 °C for 20 s, and extension at 72 °C for 30 s; and a final extension at 72 °C for 2 min.

#### Sequence analysis

After purification, the PCR products from each sample were mixed into one tube. Then, with the exception of Illumina’s Nextera prep, library preparation generally entailed: (i) A-tailing of the 3′ ends to facilitate ligation to sequencing adapters, (ii) ligation of indexed adapters, and (iii) ten PCR cycles to enrich for products with adapters ligated to both ends. Subsequent amplicon sequencing using Illumina miseq PE300 mode was carried out by the Zhejiang Dian diagnostics Co. Ltd.

From the sequencing reactions, 0.04 Mb of read data per sample was obtained, and were assessed using stringent bioinformatic filters. The resultant 560× average target coverage meant that 100% of the amplicons had at least 30× coverage. Sequence reads were aligned to the reference human genome (UCSC hg19) using the BWA software (bio-bwa.sourceforge.net). Sequence variation annotation was performed using the GATK pipeline (https://software.broadinstitute.org/gatk) and annotated using ANNOVA (annovar.openbioinformatics.org), which comprises gene annotations from multiple sources (including RefSeq, dbSNP 138, Clinvar, and 1000genome).

Mutation hotspots in *SLC12A3* were detected using next generation sequencing (NGS) and subsequently confirmed using Sanger sequencing.

## Results

### Laboratory parameters and treatment of the proband

Primary laboratory results of the proband are summarized in Table 1. Laboratory test showed severe hypokalemia, hypomagnesemia, hypocalciuria, and metabolic alkalosis, along with elevated renin activity. The electrocardiogram suggested sinus tachycardia (107 beats per minute), high voltage of the left ventricle, and ST-segment change of the anterolateral wall. An echocardiogram showed thickening of the left ventricular wall. The adrenal CT scan was unremarkable. The patient was diagnosed as GS based on her clinical phenotype. After the diagnosis, treatment with supplements of magnesium and potassium were introduced. She received 10% potassium chloride oral solution 20 ml taken orally twice daily, spironolactone 20 mg taken orally three times daily, and potassium magnesium aspartate 1 tablet taken orally three times daily. However, the electrolyte disturbances did not resolve completely, given the fact that treatment could only increase the level of potassium and magnesium up to the lower limit of the normal range. The patient had been followed up as outpatient at our endocrinology clinic for over two years since she was discharged. She denied any episode of tetany after accepting the treatment.

**Table 1:** Biochemical characteristics of the proband

Variable	Test Value	Reference range
Blood tests		
Potassium (mmol/L)	2.7^a^	3.5–5.3
Sodium (mmol/L)	138	137–147
Chloride (mmol/L)	93^a^	99–110
Calcium (mmol/L)	2.45	2.20–2.70
Phosphate (mmol/L)	1.39	0.80–1.60
Magnesium (mmol/L)	0.57^a^	0.60–1.10
Urea nitrogen (mmol/L)	3.26	2.50–7.14
Creatinine (μmol/L)	51	40–115
Fasting blood glucose (mmol/L)	4.33	4.16–5.83
OGTT-2 h Blood glucose (mmol/L)	9.58^a^	3.89–7.77
Uric acid (μmol/L)	597^a^	137–393
Renin (μg/L·h)	3.79^a^	0.13 – 1.94
Angiotensin (ng/L)	77.73^a^	23–75
Aldosterone (ng/L)	82.9	30–170
Arterial blood gas analysis		
pH	7.457	7.350–7.450
Bicarbonate (mmol/L)	29.3	21.4–24.8
24-h Urine tests		
Calcium (mmol/d)	0.5 ^a^	2.5–7.5
Potassium (mmol/d)	62.63^a^	≤ 25
Magnesium (mmol/d)	1.35^a^	2.1–8.19
Sodium (mmol/d)	78	130–261
Chloride (mmol/d)	124	177–255
Uric acid (μmol/d)	2170	1480–4430
Spot urine test		
Calcium/creatinine (mmol/mmol)	0.009^a^	

^a^ Abnormal values, urinary calcium/creatinine ratio < 0.2 is defined as hypocalciuria in GS. OGTT, oral glucose tolerance test

### Laboratory results of other family members

The serum potassium, sodium, chloride, calcium, and magnesium levels of the other family members were unremarkable.

### Gene detection results

**Fig. 1:**
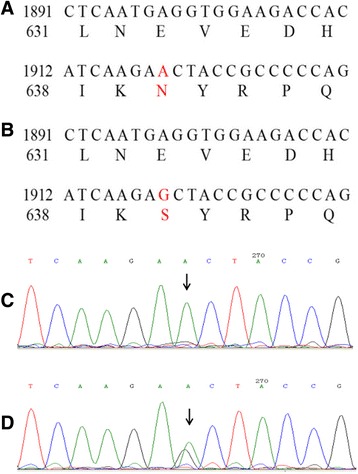
Sequencing diagram of *SLC12A3* gene Exon 15. **a** Exon 15 wild-type (DNA and amino acid sequences, GRCh37.p13); (**b**) Exon 15 heterozygous mutation type, A1919G, AAC → AGC, Asn640Ser (N640S); (**c**) Exon 15 wild-type (Sanger sequencing); (**d**) Exon 15 heterozygous mutation type (Sanger sequencing)

**Fig. 2:**
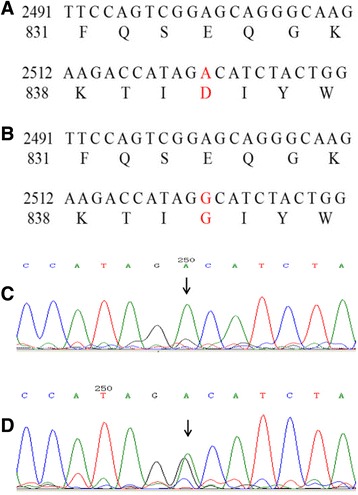
Sequencing diagram of *SLC12A3* gene Exon 21. **a** Exon 21 wild-type (DNA and amino acid sequences, GRCh37. p13); (**b**) Exon 21 heterozygous mutation, A2522G, GAC → GGC, Asp841Gly (D841G); (**c**) Exon 21 wild-type (Sanger sequencing); (**d**) Exon 21 heterozygous mutation type (Sanger sequencing)

**Fig. 3:**
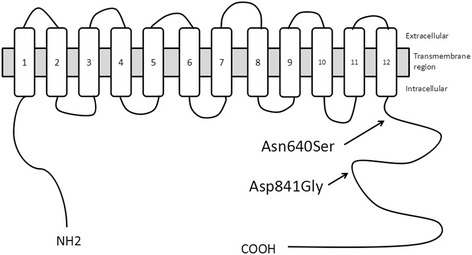
Predicted topological localization of Na-Cl cotransporter (NCC) mutations. Schematic representation of NCC protein with the intercellular N- and C-terminal domains and with squares 1 to 12 indicating the 12 transmembrane segments.^[^9^]^ Arrows indicate the position of the selected mutations

**Fig. 4:**
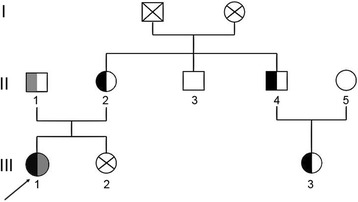
Pedigree chart of the proband’s family. The arrow indicates the proband. Filled symbols indicate subjects affected by gene mutations (c.1919A > G is marked as black and c.2522A > G is marked as grey). Men are indicated by squares, and women are indicated by circles. III1, the proband, III2, sister, III3, cousin, II1, father, II2, mother, II3, elder uncle, II4, younger uncle

DNA sequencing identified two novel missense variants in *SLC12A3* (c.1919A > G and c.2522A > G) in the proband, which resulted in substitution of Asparagine (N) 640 by Serine (S) and of Aspartic acid (D) 841 by Glycine (G), respectively (Fig. 1 and Fig. 2). Moreover, her mother (II2), uncle (II4), and cousin (III3) were the carriers of one variant (p.N640S); whereas her father (II1) was the carrier of the other (p.D841G). No *SLC12A3* gene variants were observed in other pedigree members. Both variants were located in the intracellular carboxyterminal domain of the NCC protein (Fig. 3). The pedigree analysis is summarized in Table 2 and Fig. 4. After comparing them with genomic sequence in the Ensemble database (GRCh37.p13), we concluded that this was the first report of these two pathogenic variants.

**Table 2:** Summary of *SLC12A3* variants

Subject	Mutation	Nucleotide Change	Predicted Effect	Exon	Location (GRCh37)
Proband	p.N640S	c.1919A>G AAC-AGC	CoHet, Missense	15	Chromosome 16: 56,919,270
	P.D841G	c.2522A>G GAC-GGC	CoHet, Missense	21	Chromosome 16: 56,926,940
Mother, Younger uncle, Cousin	p.N640S	c.1919A>G AAC-AGC	Het, Missense	15	Chromosome 16: 56,919,270
Father	P.D841G	c.2522A>G GAC-GGC	Het, Missense	21	Chromosome 16: 56,926,940

CoHet, compound heterozygous variant; Het, heterozygous variant

### Three-dimensional structure prediction of NCC

**Fig. 5:**
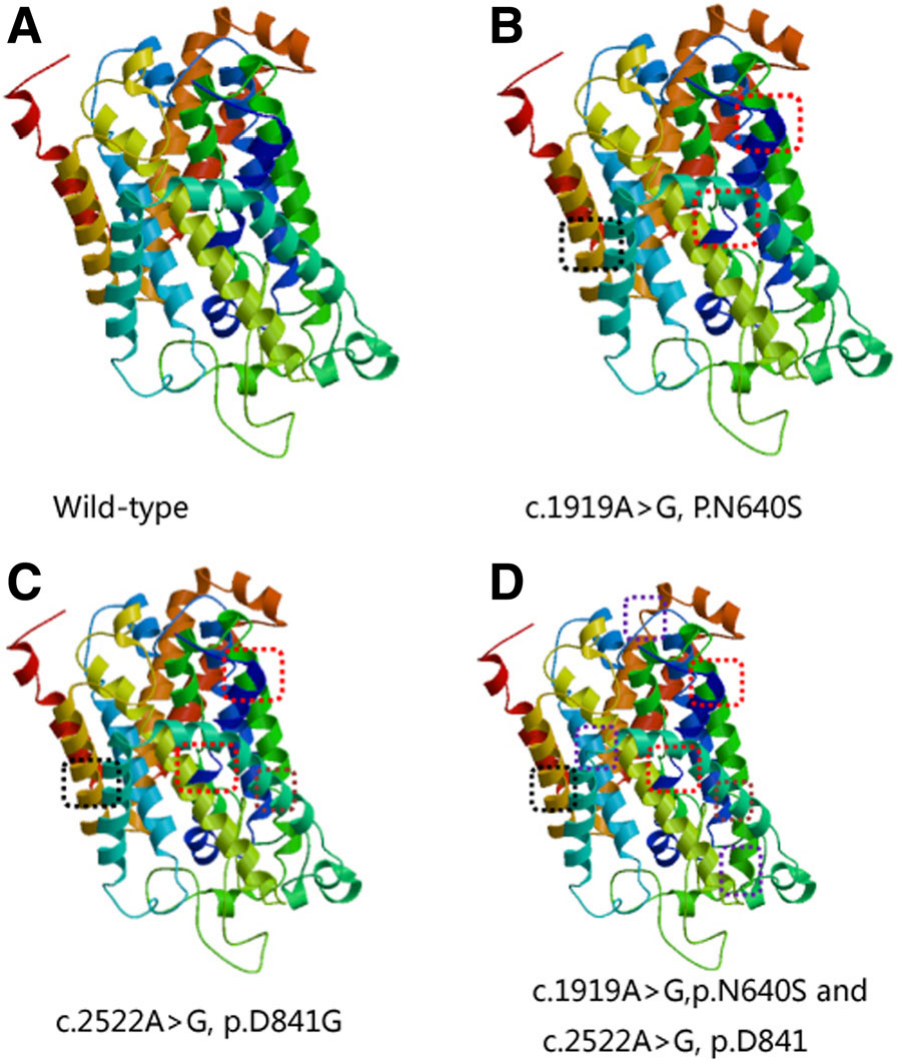
Effect of *SLC12A3* mutations on the modeled structure of the Na-Cl cotransporter (NCC) protein. Compared to the wild-type (**a**), c.1919A > G (**b**) alone caused three visible differences in the protein structure (marked with red and black frames), whereas c.2522A > G (**c**) alone led to one further difference (marked with a brown frame). Coexistence of c.1919A > G and c.2522A > G (**d**) resulted in three more differences (marked with purple frames)

We used the SWISS-MODEL workspace (http://swissmodel.expasy.org) to characterize the effect of the novel variants (c.1919A > G and c.2522A > G) on the protein structure of NCC, which demonstrated that the alterations resulting from the mutations altered the protein structure.

c.1919A > G alone caused three visible differences in the protein structure (marked with red and black frames in fig. 5) whereas c.2522A > G alone led to one further difference (marked with a brown frame). Coexistence of c.1919A > G and c.2522A > G resulted in three more differences (marked with purple frames).

## Discussion

Gitelman et al. [1] first described three female patients considered as Bartter syndrome by their clinical phenotype. These patients, however, also presented with hypocalciuria and hypomagnesemia. Thereafter, it was perceived that GS was a subtype of Bartter syndrome, but with less pronounced clinical manifestations. Simon et al. [8] further cloned the cDNA of *SLC12A3* (encoding NCC) in 1996 and revealed the molecular basis of GS, which is genetically distinct from Bartter syndrome.

NCC is mainly expressed in the epithelial cells of the distal tubule, which can be blocked by thiazide diuretics to enhance Na^+^ excretion [9]. The various genetic defects of *SLC12A3* result in NCC dysfunction, producing a clinical disorder that presents with activation of the renin-angiotensin-aldosterone system together with secondary potassium-wasting and metabolic alkalosis.

In addition to hypokalemia, metabolic alkalosis, and increased renin activity, the patient in our case also presented with hypomagnesemia and hypocalciuria, which are two indispensable criteria for diagnosing GS. Intriguingly, the mechanisms responsible for hypomagnesemia and hypocalciuria in patients with GS remain elusive. The serum magnesium balance is maintained via both intestinal absorption and renal excretion. Distal reabsorption of magnesium mainly occurs in the renal DCT via several transcellular transporters, among which the transient receptor potential channel melastatin subtype 6 (TRPM6), located at the luminal membrane, is responsible for magnesium entry from the tubular lumen into the DCT cells [10]. Nijenhuis et al. [11] pointed out that chronic thiazide administration facilitated magnesium excretion, as well as specifically reducing TRPM6 expression. Moreover, NCC-knockout (NCC−/−) mice exhibited a severe decline in the expression levels of TRPM6 [11] and widespread atrophy of DCT cells [12]. Nijenhuis also hypothesized that hyperaldosteronism might contribute to the downregulation of TRPM6. Accordingly, the significant reduction in the DCT plasma membrane area and TRPM6 downregulation could participate in the pathogenesis of hypomagnesemia.

Meanwhile, the mechanisms responsible for hypocalciuria remain controversial. The majority of calcium reabsorption occurs in the proximal convoluted tubule (PCT) and the thick ascending limb of the loop of Henle through passive diffusion, in which process, approximately 65 and 20% of calcium is reabsorbed in these sites, respectively. About 10–15% of filtered calcium reabsorption occurs through active transport in the DCT, during which period the gate keepers are the transient receptor potential cation channel subfamily V member 5 (TRPV5) and member 6 (TRPV6) [13]. Nijenhuis et al. [11] proved that hypovolemia enhanced proximal calcium reabsorption, which in turn, elevated the electrochemical gradient and drove passive calcium transport in the PCT. However, given the fact that NCC_S707X/S707X_ mice exhibited increased expression levels of TRPV5 and TRPV6 in the DCT, Yang et al. [14] proposed that enhanced distal tubular calcium reabsorption might also be involved in the pathogenesis of hypocalciuria in patients with GS.

In our case, the patient also suffered from metabolic complications, such as hyperuricemia and impaired glucose tolerance. Thiazide diuretics can elevate serum uric acid (UA) by up to 35% [15]. There are multiple putative mechanisms for thiazide-induced hyperuricemia. It was reported that volume contraction created by diuretic treatment could lead to a consequent decrease in UA excretion [16]. Reduction of multidrug resistance protein 4 (MRP4)-mediated UA efflux via thiazides was also a causal factor [17]. Moreover, it was identified that UA reuptake was elevated through human organic anion transporter 4 (hOA4)-mediated exchange with hydrochlorothiazide (HCTZ) [18]. In experimental hyperuricemic rats, UA caused impairment of endothelial function, resulting in the development of insulin resistance. However, hyperinsulinemia was significantly reversed by lowering UA levels using Benzbromarone, a uricosuric agent [19]. Later, researchers discovered that HCTZ treatment of fructose-induced rats aggravated hyperuricemia and hyperglycemia, and insulin resistance was improved by potassium supplementation and correcting hyperuricemia with Allopurinol [20]. Meanwhile, according to 59 thiazide diuretic clinical trials, glucose intolerance seemed to have a strong relationship with hypokalemia. However, if thiazide-induced hypokalemia was corrected promptly, it was possible to rescue glucose intolerance and, therefore, prevent the development of diabetes [21]. Rowe et al. [22] further supported this idea by proving that hypokalemia could result in impaired glucose tolerance secondary to impaired insulin secretion by establishing an experimentally generated hypokalemic state. Therefore, it was deduced that hyperuricemia and hypokalemia are both contributing factors for impaired glucose tolerance.

The proband, her mother, and two uncles were diagnosed as HCM for many years, which is an autosomal dominant inheritance disease caused by mutations in genes encoding sarcomeric proteins [23]. Electrocardiogram and echocardiography of the proband confirmed the diagnosis of HCM. The electrocardiogram suggested tachycardia, which might have resulted from hypokalemia; however, the proband denied any episode of palpitation. Unfortunately, the proband’s sister (III2) was never evaluated for HCM before her sudden death. Hypokalemia caused by GS could induce adverse cardiac events, including all kinds of arrhythmias. It was reported that patients with GS could suffer from sudden cardiac arrest [5, 24]. Therefore, we considered it was reasonable to attribute her sudden death to both HCM and GS, because she had a 50% probability of suffering from HCM and a 25% probability of suffering from GS according to the genetic analysis of their parents.

The clinical manifestations in patients with GS are extremely variable, not only in patients carrying different *SLC12A3* mutations [25], but also in unrelated patients carrying the same mutation [26], and even in affected subjects from the same family [5, 27]. Currently, the underlying molecular mechanisms of phenotype heterogeneity in patients with GS are not fully understood. After analyzing the transcription and function of SLC12A3, researchers presumed that the potential severity of GS was related to the nature of the *SLC12A3* mutations together with male gender [5].

To date, 488 mutations of the *SLC12A3* gene have been discovered in patients with GS (HGMD, professional 2017.1), including missense mutations, shear mutations, nonsense mutations, and frame shift mutations, among which missense mutations are the most commonly described. Moreover, compound heterozygous mutations are more common than homozygous mutations [28].

Researchers screened for *SLC12A3* mutations in 448 patients suspected of having GS using direct sequencing of genomic DNA. They identified two mutations in 315 patients (70%); one mutation in 81 patients (18%); and no mutation in 52 patients (12%). Among patients suspected of having GS, 18–40% carried only one mutant allele; therefore, researchers further screened for large rearrangements, which are often missed by direct sequencing. The results demonstrated that large rearrangements accounted for approximately 6% of the mutations in GS [29].

To the best of our knowledge, we identified the compound heterozygous variants (c.1919A > G, p.N640S and c.2522A > G, p.D841G) for the first time. The patient’s mother, younger uncle, and cousin were the carriers of one variant (c.1919A > G), and her father was the carrier of the other (c.2522A > G). However, the patient was the only member of the pedigree presenting with apparent clinical manifestations and suffering from GS. Such cases have been reported before [30, 31]. In these studies, the probands carried compound heterozygous variants, with one mutant inherited from their mother and the other from their father. However, their parents were both healthy.

The modeled structure predicted by the Swiss-Model Workspace suggested that c.1919A > G alone caused three visible differences in the protein structure and that c.2522A > G alone caused one more difference. Furthermore, coexistence of c.1919A > G and c.2522A > G resulted in three more differences compared with c.2522A > G alone. Thus, we hypothesized that the presence of more mutations would cause more structural alterations leading to protein dysfunction. However, further functional analyses are needed to establish the relationship between the genotype and phenotype.

How do these mutations lead to protein dysfunction? Previous functional research proposed at least five different underlying mechanisms involving the reduced or abolished transport activity, which could be summarized as follows: (1) impaired protein synthesis; (2) defective protein processing; (3) defective protein insertion; (4) protein dysfunction with impaired intrinsic activity; and (5) accelerating the degradation of protein [32, 33].

NCC is a 1030-amino-acid integral membrane protein with 12 transmembrane domains and intercellular N- and C-terminal domains (Fig. 3). The two mutations identified in our study are located in the C-terminal domain. Lemmink et al. [34] hypothesized that the intercellular C-terminal domain was a hot spot for *SLC12A3* mutations because 14 of the 20 mutations identified in their study were located in this region. The C-terminal end contains three acknowledged protein kinase phosphorylation sites. Given that these sites are indispensable to maintaining the protein’s function, and regulate many ion transport systems, it is reasonable to suppose that mutations in this domain might alter the protein configuration to interfere with phosphorylation, resulting in defective NCC activity.

## Conclusions

In this study, two novel missense variants (N640S and D841G) located in the C-terminal domain of the NCC protein were identified in the proband. One variant (N640S) was inherited from her mother and the other (D841G) was inherited from her father. In view of their locations, we presumed that these two novel variants would cause impairment or loss of NCC function. Further functional studies are warranted to investigate the molecular basis of the defective NCC function caused by these two possible pathogenic variants.

## Additional files

Additional file 1: Table S1. Clinical and biochemical characteristics of all five family members. Clinical and biochemical characteristics of all five family members are listed in Table S1. (DOCX 14 kb)

Additional file 2: Table S2. Primer sequences of the *SLC12A3* gene and annealing temperature of each pair. Thirty-three primer pairs designed to amplify the coding sequence of the *SLC12A3* gene, and annealing temperature of each pair, are provided in Table S2. (DOCX 14 kb)

## Abbreviations

CT: Computed tomography
DCT: Distal convoluted tubule
GS: Gitelman syndrome
HCM: Hypertrophic cardiomyopathy
HCTZ: Hydrochlorothiazide
MRP4: Multidrug resistance protein 4
NCC: Na-Cl cotransporter
NGS: Next generation sequencing
OA4: Organic anion transporter 4
PCR: Polymerase chain reaction
PCT: Proximal convoluted tubule
RAAS: Renin-angiotensin-aldosterone system
SLC12A3: Solute carrier family 12 member 3
TRPM6: Transient receptor potential channel melastatin subtype 6
TRPV5 and 6: Transient receptor potential cation channel subfamily V member 5 and member 6
UA: Uric acid
