# Supplementary material for: A novel compound heterozygous variant of the SLC12A3 gene in Gitelman syndrome pedigree
Source: BMC Med Genet. 2018 Jan 29;19:17. doi: 10.1186/s12881-018-0527-7 (PMC5789536; doi:10.1186/s12881-018-0527-7)
Supplement: Supplementary file 1 — Clinical and biochemical characteristics of all five family members. Clinical and biochemical characteristics of all five family members are listed in Table S1. (DOCX 14 kb) [file 12881_2018_527_MOESM1_ESM.docx]

| Subject | Gender | Age | Medical history | Blood pressure (mmHg) | Heart rate (beats/min) | Potassium (mmol/L) | Sodium (mmol/L) | Chloride (mmol/L) |
| --- | --- | --- | --- | --- | --- | --- | --- | --- |
| Mother | female | 60 | HCM | 126/73 | 88 | 3.9 | 139 | 102 |
| Older uncle | male | 55 | HCM | 130/78 | 86 | 4.1 | 141 | 104 |
| Younger uncle | male | 52 | HCM | 134/80 | 84 | 3.8 | 140 | 105 |
| Cousin | female | 24 | No | 104/65 | 80 | 4.2 | 139 | 103 |
| Father | male | 62 | No | 128/70 | 78 | 3.8 | 137 | 100 |

HCM, Hypertrophic cardiomyopathy.
