# Supplementary material for: A novel compound heterozygous variant of the SLC12A3 gene in Gitelman syndrome pedigree
Source: BMC Med Genet. 2018 Jan 29;19:17. doi: 10.1186/s12881-018-0527-7 (PMC5789536; doi:10.1186/s12881-018-0527-7)
Supplement: Supplementary file 2 — Primer sequences of the SLC12A3 gene and annealing temperature of each pair. Thirty-three primer pairs designed to amplify the coding sequence of the SLC12A3 gene, and annealing temperature of each pair, are provided in Table S2. (DOCX 14 kb) [file 12881_2018_527_MOESM2_ESM.docx]

| **Name** | **Primer Sequence (5'-3')** | | **Annealing Temp(℃）** |
| --- | --- | --- | --- |
|  | **Forward** | **Reverse** |  |
| SLC12A3-1 | CGCAGCCTATAAAACCACCC | GTTAGGTAGCTGGGTCGAGG | 60 |
| SLC12A3-2 | TTAAAAGCCCCTCAAGCAGC | CCGGCAGGCTTTAATCTATTGT | 60 |
| SLC12A3-3 | ACCCTCTTGCCCCATAGAAC | AGGGGCCTGAGACTGAACC | 57 |
| SLC12A3-4 | TCCCAAAGTGACAGAGACCC | GGGCAGGAGATAGCTGAGG | 57 |
| SLC12A3-5 | GGCTGTCTACACCACGAGAT | CAGTGATCAGGCTGTGGTTC | 60 |
| SLC12A3-6 | TTAGAGCCACACTGTCCAGG | AGATGGTACCACTGCACTCC | 60 |
| SLC12A3-7 | TTTGGCGGTCTTGTTCACTG | TGGAAAAGCAAGGCCTGATG | 57 |
| SLC12A3-8 | GGAATCTGGCCCATTGTGTG | GAGCTGAGTTGGACTGGACT | 57 |
| SLC12A3-9 | CCTTCCTCTCTGTGCTGGAA | TTCACATCCTAAGGTCGCCA | 60 |
| SLC12A3-10 | TAATGAAGGGACAGCTGCCC | GAACCCCTCTCTCCTAAGCC | 57 |
| SLC12A3-11 | GGTGGACACTGGGATCTCC | TGCAGGTGTTTGTCAAGTCG | 57 |
| SLC12A3-12 | TGTCTTTATGGTAGGGCGGG | TCACCCCTCAAGACTTAGCC | 57 |
| SLC12A3-13 | GACACCATCCCTTTGAGCTTC | TGCTAGGATTACAGGCATGAG | 57 |
| SLC12A3-14 | TCATGGCTGGTGGGTACAG | GATAAAGGGGAGGGGCAGAG | 57 |
| SLC12A3-15 | AACTTTTCAACGTGCAGCCA | TCCTCCATGTCTGTTCCCTC | 57 |
| SLC12A3-16 | CCAAAAGAGCAGGAAGAGCC | CACTCTGTGGGTGGACATCA | 60 |
| SLC12A3-17 | TCTGGGCAAAAGAAAAGGGC | GCTGACATCACACCATTGCA | 57 |
| SLC12A3-18 | TCAGAAAGTTGGGGCTCTGG | GCCTCAGCGTGTTTCTTCTT | 60 |
| SLC12A3-19 | AGTCCTGGGGTACTCTCCAA | TGGGAAAACTGATGGGCTCT | 57 |
| SLC12A3-20 | ACTGGAATGTGAGGAGCCG | AATCTTTGACACGGCCAAGC | 57 |
| SLC12A3-21 | CCGGGAGAGAGAAGCTGAAA | AACTAGAGGCCAGACAGAGG | 57 |
| SLC12A3-22 | TAAACCCTACCCCACAGAGC | TGGAATGGAGAGAAACCTGCT | 57 |
| SLC12A3-23 | TGGCACACATCTGTAATCCCA | AGAAGTTGTGCCCTCTGACA | 57 |
| SLC12A3-24 | TAAATGGCAGTGTCCGATGG | CATCAGCCTGAACGACTGTG | 57 |
| SLC12A3-25 | TCTTGGAGGAGGTGAGCTTG | CATGCTAAGAATCGCCAGGG | 57 |
| SLC12A3-26 | GCCCCGTGGTAATCTCTCTT | GAGTTTTCCTTGCAGCTCCA | 57 |
| SLC12A3-27 | ATCTGGCTGGACTCCACTTC | GAAATGCTTGAACCCGGGAG | 60 |
| SLC12A3-28 | CAGGCTAGGGTGTAATGGCA | GCTGCAAGTCTCAGTGTGAA | 60 |
| SLC12A3-29 | TGGTTCTTTGGACGAGGGAC | CCCTTGCCACTAGACTCCTT | 57 |
| SLC12A3-30 | ACACGTGAGGAGGGGTATTG | ATTCGATGGCAGTGCTCTTG | 57 |
| SLC12A3-31 | CACGTTGAACATCCCCAGTG | CAGACAGTGGGGCTTCAGAG | 60 |
| SLC12A3-32 | GTCATCCCACTGCAAAGCC | CCGTTGACTGAATGACCACC | 57 |
| SLC12A3-33 | GCCTTTCTAGAGCCACGTAG | AAATGGTTCCCCGTGAGTCT | 60 |
